# Honokiol ameliorates oxidative stress-induced DNA damage and apoptosis of c2c12 myoblasts by ROS generation and mitochondrial pathway

**DOI:** 10.1080/19768354.2019.1706634

**Published:** 2019-12-28

**Authors:** Cheol Park, Sung Hyun Choi, Jin-Woo Jeong, Min Ho Han, Hyesook Lee, Su Hyun Hong, Gi-Young Kim, Sung-Kwon Moon, Wun-Jae Kim, Yung Hyun Choi

**Affiliations:** a Department of Molecular Biology, College of Natural Sciences, Dong-eui University, Busan, Republic of Korea; b Department of System Management, Korea Lift College, Geochang, Republic of Korea; c Freshwater Bioresources Utilization Bureau, Nakdonggang National Institute of Biological Resources, Sangju, Republic of Korea; d National Marine Biodiversity Institute of Korea, Seocheon, Republic of Korea; e Department of Biochemistry, Dong-eui University College of Korean Medicine, Busan, Republic of Korea; f Anti-Aging Research Center, Dong-eui University, Busan, Republic of Korea; g Department of Marine Life Sciences, Jeju National University, Jeju, Republic of Korea; h Department of Food and Nutrition, Chung-Ang University, Anseong, Republic of Korea; i Department of Urology, College of Medicine, Chungbuk National University, Cheongju, Republic of Korea

**Keywords:** Honokiol, oxidative stress, DNA damage, apoptosis, ROS

## Abstract

Honokiol is one of the main active components of *Magnolia officinalis*, and has been demonstrated to have multiple pharmacological activities against a variety of diseases. Recently, this phenolic compound is known to have antioxidant activity, but its mechanism of action remains unclear. The purpose of the current study was to evaluate the preventive effects of honokiol against oxidative stress-induced DNA damage and apoptosis in C2C12 myoblasts. The present study found that honokiol inhibited hydrogen peroxide (H_2_O_2_)-induced DNA damage and mitochondrial dysfunction, while reducing reactive oxygen species (ROS) formation. The inhibitory effect of honokiol on H_2_O_2_-induced apoptosis was associated with the up-regulation of Bcl-2 and down-regulation of Bax, thus reducing the Bax/Bcl-2 ratio that in turn protected the activation of caspase-9 and -3, and inhibition of poly (ADP-ribose) polymerase cleavage, which was associated with the blocking of cytochrome *c* release to the cytoplasm. Collectively, these results demonstrate that honokiol defends C2C12 myoblasts against H_2_O_2_-induced DNA damage and apoptosis, at least in part, by preventing mitochondrial-dependent pathway through scavenging excessive ROS.

## Introduction

Honokiol (2-(4-hydroxy-3-prop-2-enyl-phenyl)-4-prop-2-enylphenol) is a naturally occurring biphenolic compound derived from the medicinal plant *Magnolia officinalis*, which have been widely used in traditional medicine (Lee et al. 2011). Many previous studies showed that honokiol had advantageous multi-pharmacological effects, including antioxidant activity. For example, improvement of focal cerebral ischemia-reperfusion injury in the rat brain by honokiol was due to the inhibition of lipid peroxidation and reduction of neutrophil activation/infiltration through interference with the reactive oxygen species (ROS) production (Liou et al. 2003). Similarly, blockade of oxidized low-density lipoprotein-induced mitochondrial mediated apoptosis by honokiol in vascular endothelial cells was associated with the inhibition of ROS production (Ou et al. 2006).

Although adequate levels of ROS activate important signaling pathways, chronic, persistent or persistence or excessive production of ROS can cause oxidative damage to cells. Because mitochondria represent the major sources of ROS and the most vulnerable targets of ROS, an inadequate accumulation of ROS has been recognized as one of the mechanisms leading to apoptosis associated with mitochondrial dysfunction. Moreover, the accumulation of ROS could reduce mitochondrial membrane potential (MMP), resulting in compromise of the ATP production (Rigoulet et al. 2011; Sosa et al. 2013). Subsequently, the apoptogenic factors are released into the cytoplasm from the mitochondrial intermembrane space due to the loss of MMP, and the caspase cascade is activated, which could eventually trigger apoptosis (Rigoulet et al. 2011; Sosa et al. 2013).

Similar to many other types of cells, excessive ROS produced by oxidative stress is participated in the development of numerous muscle disorders and diseases (Rodney and Pal 2016; Durgin and Straub 2018). Hydrogen peroxide (H_2_O_2_), one of the major ROS, dissociates intracellularly to form highly reactive and destructive hydroxyl radicals that contribute to DNA damage and subsequent death in muscle cells (Vara and Pula 2014; Cobley et al. 2015). Therefore, potential antioxidants can have therapeutic as well as protective effects on ROS-mediated damage to muscle cells. However, we still lack detailed insight into the mechanisms that control the defense efficacy of honokiol due to ROS production in muscle cells. The aim of this study was to explore the usefulness of honokiol for defensive against oxidative stress in muscle cells. Our findings show that honokiol effectively reduced H_2_O_2_-induced cytotoxicity through the attenuation of ROS production in cultured mouse C2C12 skeletal myoblasts.

## Materials and methods

### Cell culture and honokiol treatment

C2C12 cells obtained from the American Type Culture Collection (Manassas, VA, USA) were cultured in DMEM containing 10% fetal bovine serum and 100 U/ml penicillin and streptomycin (WelGENE Inc., Daegu, Republic of Korea) at 37°C in humidified air with 5% CO_2_. Honokiol, which was purchased from LKT Laboratories (St. Paul, MN, USA), was dissolved in dimethyl sulfoxide (DMSO, Sigma-Aldrich Chemical Co., St. Louis, MO, USA), and diluted with cell culture medium to adjust the final treatment concentrations prior to use in the experiments.

### Cell viability assay

For the cell viability study, the cells were incubated with different concentrations of honokiol for 24 h, or pre-incubated with honokiol for 1 h, before H_2_O_2_ treatment for 24 h. The cells were also treated with 10 mM of *N*-acetyl cysteine (NAC) for 1 h in the presence or absence of H_2_O_2_. Subsequently, cell viability was determined using 3-(4,5-dimethylthiazol-2-yl)-2,5-diphenyltetrazolium bromide (MTT, Sigma-Aldrich Chemical Co.) assay according to the previous study (Jeon et al. 2017).

### Measurement of ROS level

To detect ROS production, cells were treated with or without honokiol and NAC for 1 h, before adding H_2_O_2_ for a further 1 h. The cells were washed with cold phosphate buffered saline (PBS) and stained with 10 µM of 2′,7′-dichlorofluorescein diacetate (DCF-DA, Sigma-Aldrich Chemical Co.) for 20 min at 37°C. The relative fluorescence intensity of the cell suspensions was measured by a flow cytometer (Becton Dickinson, San Jose, CA, USA). For image analysis of ROS production, the cells were mounted on a microscope slide and images were visualized using a fluorescence microscope (Carl Zeiss, Oberkochen, Germany).

### Apoptosis assay

Cells were fixed with 4% paraformaldehyde for 30 min at room temperature (RT) and stained with 1.0 mg/ml of 4,6-diamidino-2-phenylindole (DAPI, Sigma-Aldrich Chemical Co.) solution for 10 min at RT, and then washed with PBS. The morphological changes in the nucleus were examined using a fluorescence microscope. To determine the extent of apoptosis by a flow cytometer using Annexin V/propidium iodide (PI) double staining, the cells were collected and then stained with fluorescein isothiocyanate (FITC)-conjugated annexin V and PI (BD Pharmingen, San Diego, CA, USA) at RT for 20 min. The fluorescence intensities of the cells were detected by a flow cytometer, and acquisition was performed using the Cell Quest Pro software.

### Internucleosomal DNA fragmentation assay

The cells were dissolved in lysis buffer [10 mM Tris-HCl (pH 7.4), 150 mM NaCl, 5 mM Na-ethylenediaminetetraacetic acid (EDTA), 0.5% Triton X-100, and 0.1 mg/ml proteinase K] for 30 min at RT. DNA from the supernatant was extracted by chloroform/phenol/isoamyl alcohol (24/25/1, v/v/v), and was precipitated by ethanol. The extracted DNA was then transferred to 1.5% agarose gel containing 0.1 µg/ml ethidium bromide (EtBr), and electrophoresis was carried out at 70 V.

### Comet assay for DNA damage

After the respective treatments, cells were mixed with 0.75% low-melting agarose (LMA), and then transferred to a microscope slide precoated with a layer of 0.75% normal-melting agarose for solidification. The slides were covered with LMA, and immersed in lysis solution [2.5 M NaCl, 100 mM Na-EDTA, 10 mM Tris, 1% Triton X-100, and 10% DMSO, pH 10] for 1 h at 4°C. The slides were then placed in a horizontal electrophoresis tank containing electrophoresis buffer (300 mM NaOH, 10 mM Na-EDTA, pH 10) for 20 min. Thereafter, electrophoresis was carried out in the same buffer for 20 min at 4°C. After electrophoresis, the slides were rinsed gently with the neutralization buffer (0.4 M Tris-HCl, pH 7.5) for 10 min at 25°C. The slides were stained with 40 µg/ml EtBr and observed under a fluorescence microscope. Single cells tail length and tail DNA percentage were analyzed by CometScore version 2.0 (TriTek, USA) software as previous described (Jamialahmadi et al. 2014).

### Western blot analysis

The cells were lysed with lysis buffer for 30 min to extract whole-cell proteins, as described in the previous study (Kim et al. 2018). In a parallel experiment, mitochondrial and cytosolic proteins were extracted using a mitochondria isolation kit (Active Motif, Carlsbad, CA, USA). The equal amounts of protein samples were subjected to sodium-dodecyl sulfate-polyacrylamide gel electrophoresis, and then transferred onto polyvinylidene fluoride membranes (Millipore, Bedford, MA, USA). Membranes were probed with primary antibodies overnight at 4°C. The membranes were then incubated with the appropriate secondary antibodies conjugated with horseradish peroxidase for 2 h at RT. The protein bands were visualized by incubating the membranes in an enhanced chemiluminescence (ECL) reagent (Amersham Biosciences, Westborough, MA, USA).

### Determination of 8-hydroxy-2′-deoxyguanosine (8-OHdG)

The BIOXYTECH® 8-OHdG-EIA^™^ kit (OXIS Health Products Inc., Portland, OR, USA) was used for the quantitative measurement of oxidative DNA damage. Briefly, the cellular DNA was isolated using the DNA Extraction Kit (iNtRON Biotechnology Inc., Sungnam, Republic of Korea), and quantified. The amount of 8-OHdG, a deoxyriboside form of 8-oxoGuanine, in the DNA was determined by calculation on a standard curve measured at 450 nm absorbance using a microplate reader (Dynatech Laboratories, Chantilly, VA, USA).

### Measurement of MMP (Δψm)

To measure the loss of MMP, the cells were incubated in media containing 10 µM of 5,5′6,6′-tetrachloro-1,1′,3,3′-tetraethyl-imidacarbocyanine iodide (JC-1, Sigma-Aldrich Chemical Co.) at 37°C for 20 min at RT. After washing twice with PBS to remove unbound dye, the green (JC-1 monomers) and red (JC-1 aggregates) fluorescence ratio that monitored the proportion of mitochondrial depolarization was immediately acquired on a flow cytometer.

### Detection of ATP levels

The levels of intracellular ATP were determined using a firefly-luciferase-based ATP Bioluminescence assay kit (Roche Applied Science, Indianapolis, IN, USA). Briefly, the cells were lysed with the provided lysis buffer, and the collected supernatants were mixed with an equal amount of luciferase agent, which catalyzed the light production from ATP and luciferin. The emitted light was immediately measured using a microplate luminometer, and the ATP level was calculated according to the ATP standard curve.

### Statistical analysis

Data were expressed as the mean ± standard deviation (SD) from at least three independent experiments. Statistical significance analysis was carried out using ANOVA-Tukey’s post hoc test. A *p*-value of less than 0.05 was considered to indicate statistical significance.

## Results

### Honokiol inhibits H_2_O_2_-induced cytotoxicity

**Figure 1.**
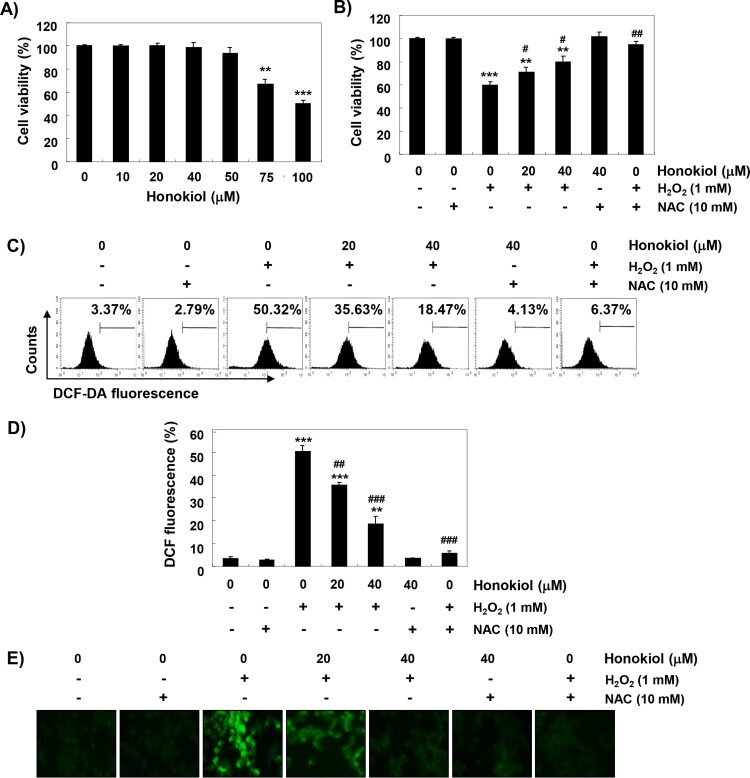
The protective effects of honokiol against H_2_O_2_-induced ROS accumulation and cytotoxicity in C2C12 cells. (A and B) C2C12 cells were treated with various concentrations of honokiol for 24 h (A) or were treated with 1 mM H_2_O_2_ for 24 h, after honokiol or NAC pre-treatment (B). The cell viability was examined by the MTT assay. The data are shown as mean ± SD obtained from three independent experiments. Statistical analyses were conducted using analysis of variance (ANOVA-Tukey’s *post hoc* test) between groups. *p < 0.05 and **p < 0.01 *vs* control group, ^#^*p *< 0.05 and ^##^*p *< 0.01 *vs* H_2_O_2_-treated group. (C-E) The cells were pretreated with honokiol or 10 mM NAC for 1 h, and then stimulated with or without 1 mM H_2_O_2_ for 1 h. (C) After staining with DCF-DA, DCF fluorescence was monitored by flow cytometer. (D) The data are shown as mean ± SD obtained from three independent experiments (**p < 0.01 and ***p < 0.001 *vs* control group, ^##^*p *< 0.01 and ^###^*p *< 0.001 *vs* H_2_O_2_-treated group). (E) The fluorescent images were obtained by a fluorescence microscope.

Figure 1(A) shows that C2C12 cells treated with concentrations of 75 µM or more showed significant decrease in cell viability, but no significant change compared to the control group was found until 50 µM. H_2_O_2_ concentration for inducing oxidative stress was selected to be 1 mM, which showed a survival rate of about 60%, compared with the control cells. To evaluate the protective effect of honokiol on H_2_O_2_-induced cytotoxicity, cells were treated with 20 and 40 mM honokiol for 1 h before treatment with H_2_O_2_, and cultured for 24 h. Figure 1(B) shows that pretreatment with honokiol significantly restored cell viability, as compared to H_2_O_2_ alone. In addition, the H_2_O_2_-induced decrease in cell viability was completely suppressed to the control level in the cells pretreated with NAC, a positive ROS scavenger (Figure 1(B)).

### Honokiol attenuates H_2_O_2_-induced ROS generation

Next, we investigated whether the protective effects of honokiol on H_2_O_2_-induced cytotoxicity were due to the blockade of oxidative stress. As shown in Figure 1(C,D), the ROS generation was significantly increased within 1 h in the cells exposed to H_2_O_2_ compared to the control; however, H_2_O_2_-induced accumulation of ROS in cells pretreated with honokiol was significantly reduced. In the fluorescence microscope observation, we further confirmed that honokiol had a powerful ROS scavenging effect (Figure 1(E)). Also, the production of ROS by H_2_O_2_ was greatly blocked by pretreatment of NAC, and the degree of ROS generation was not significantly changed in the honokiol alone group.

### Honokiol suppresses H_2_O_2_-induced apoptosis

**Figure 2.**
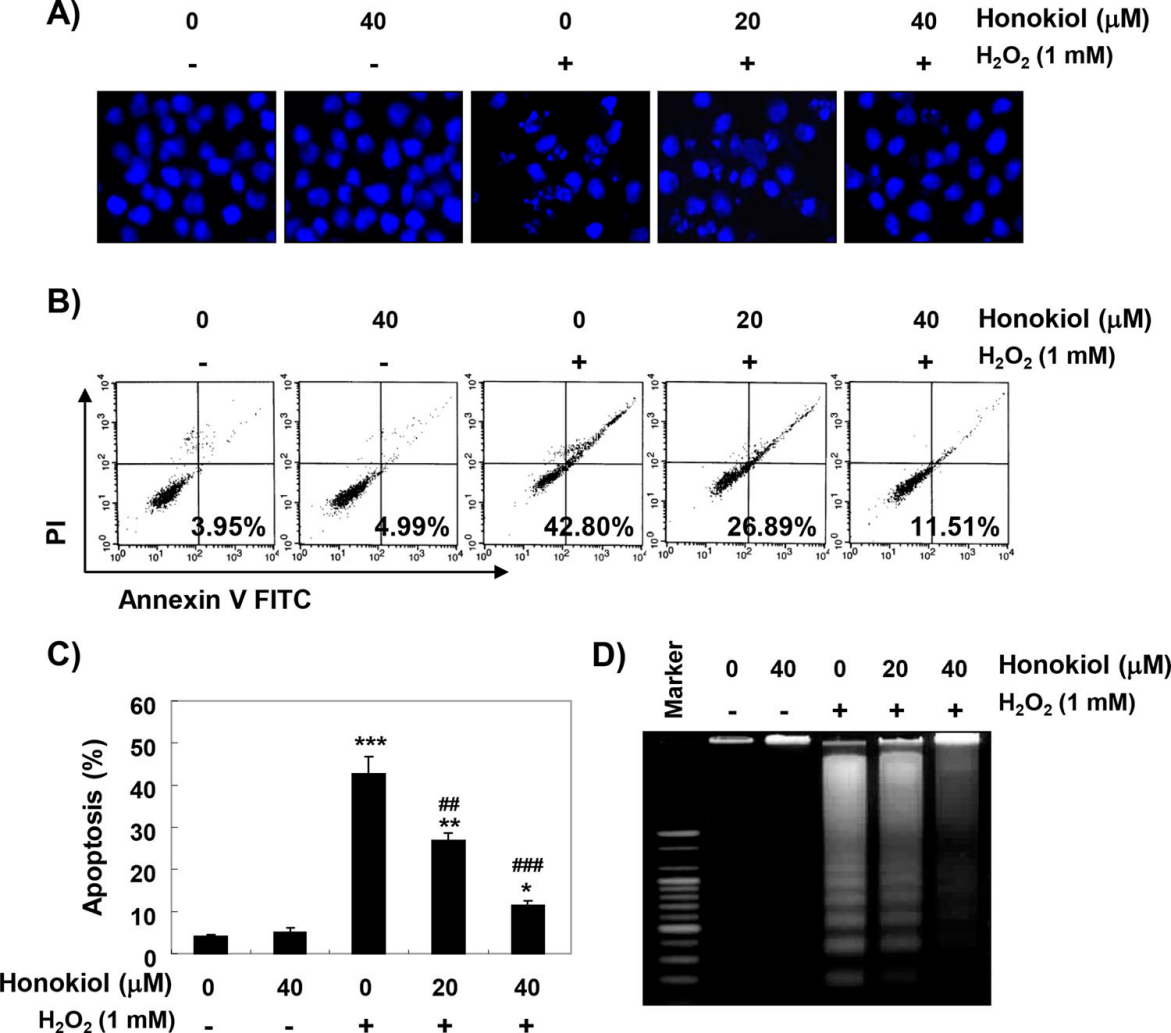
Attenuation of H_2_O_2_-induced apoptosis by honokiol. Cells were treated with honokiol for 1 h, and then stimulated with or without 1 mM H_2_O_2_ for 24 h. (A) The cells were collected, fixed, and stained with DAPI solution. The stained nuclei were pictured under a fluorescence microscopye. (B and C) The cells cultured under the same conditions were collected, and stained with FITC-conjugated Annexin V and PI for flow cytometry analysis. (B) The percentages of apoptotic cells were determined by counting the percentage of Annexin V-positive cells. (C) Data were expressed as the mean ± SD of three independent experiments. Statistical analyses were conducted using analysis of variance (ANOVA-Tukey’s *post hoc* test) between groups. *p < 0.05, **p < 0.01 and ***p < 0.001 *vs* control group, ^##^*p *< 0.01 and ^##^*p *< 0.001 *vs* H_2_O_2_-treated group. (D) DNA fragmentation was analyzed by extracting genomic DNA, electrophoresis in 1.5% agarose gel, and then visualizing by EtBr staining.

DAPI staining, flow cytometry, and agarose gel electrophoresis analysis were performed, to investigate whether the cytoprotective effect of honokiol against H_2_O_2_ was related to apoptosis suppression. The fluorescent images in Figure 2(A) revealed that the control cells had intact nuclei, while the H_2_O_2_-treated cells showed significant chromatin condensation. However, the morphological changes were markedly attenuated in the cells pretreated with honokiol before the treatment with H_2_O_2_. The results of Annexin V/PI double staining also showed that the pretreatment of honokiol significantly decreased the frequency of apoptotic cells in H_2_O_2_-stimulated cells (Figure 2(B,C)). In addition, the agarose gel electrophoresis result showed that H_2_O_2_-induced DNA fragmentation was completely attenuated by the pretreatment of honokiol (Figure 2(D)).

### Honokiol reduces H_2_O_2_-induced DNA damage

**Figure 3.**
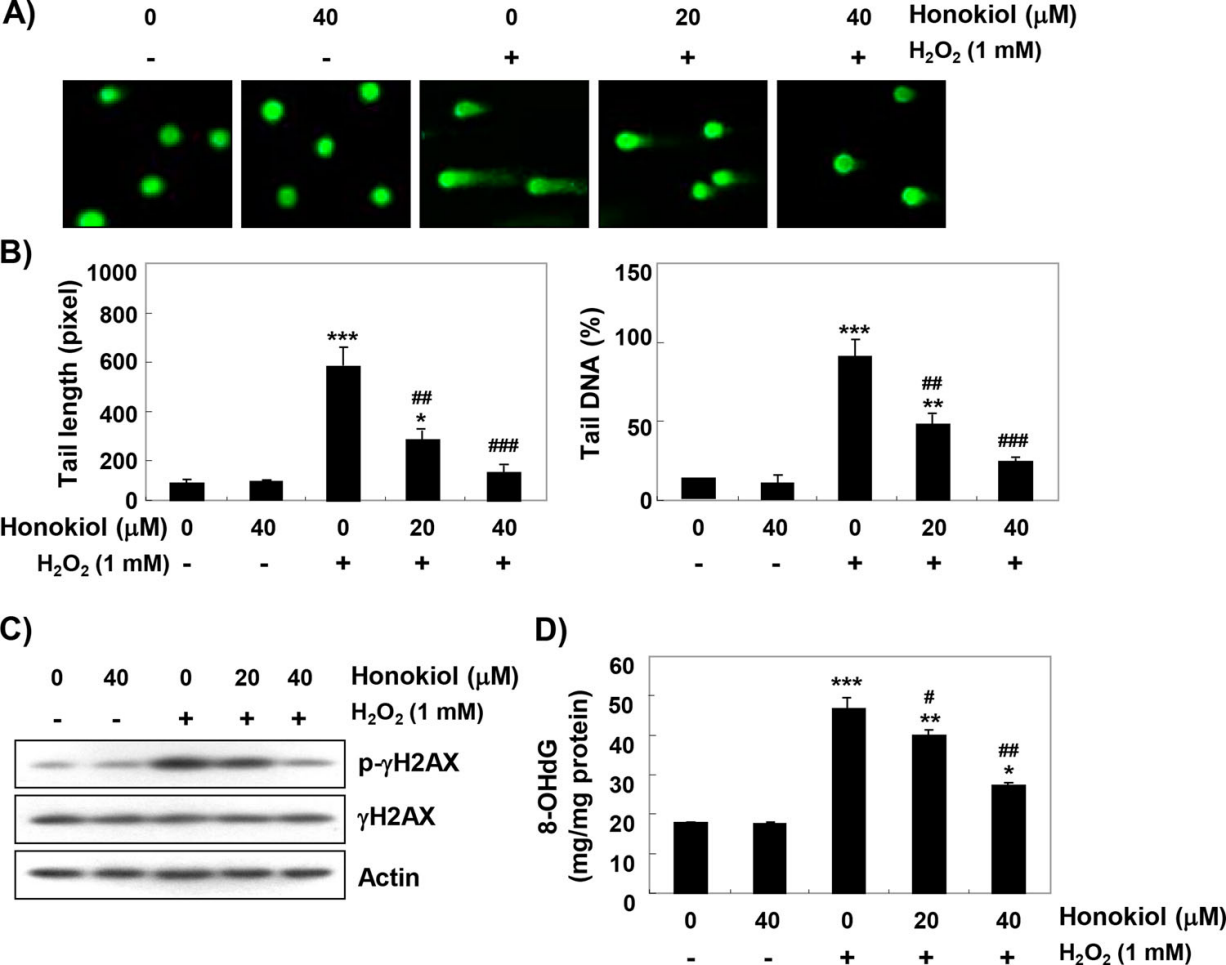
Protection of H_2_O_2_-induced DNA damage by honokiol. Cells were pretreated with honokiol for 1 h, and then stimulated with or without 1 mM H_2_O_2_ for 24 h. (A) The comet assay was performed, and representative images of comet assay were taken. (B) The statistical analysis of tail length and tail DNA percentage. Statistical analyses were conducted using analysis of variance (ANOVA-Tukey’s *post hoc* test) between groups. *p < 0.05, **p < 0.01 and ***p < 0.001 *vs* control group, ^##^*p *< 0.01 and ^###^*p *< 0.001 *vs* H_2_O_2_-treated group. (C) The cellular proteins were prepared, and p-γH2AX and γH2AX protein levels were assayed by Western blot analysis. (D) The amount of 8-OHdG in DNA was determined using an 8-OHdG-EIA kit. The measurements were made in triplicate, and values are expressed as the mean ± SD. Statistical analyses were conducted using analysis of variance (ANOVA-Tukey’s *post hoc* test) between groups. *p < 0.05, **p < 0.01 and ***p < 0.001 *vs* control group, ^#^*p *< 0.05 and ^##^*p *< 0.01 *vs* H_2_O_2_-treated group.

To determine whether honokiol prevents DNA damage, the comet assay was performed. Figure 3(A,B) shows that similar to the control cells, the smeared pattern of nuclear DNA was not observed in cells treated with honokiol alone. However, there was a clear increase in the length of tail in H_2_O_2_-treated cells. On the other hand, in honokiol-pretreated cells, tail length and tail DNA were obviously shortened. Additionally, the immunoblotting results showed a marked increase in γH2AX phosphorylation (at serine 139, p-γH2AX) in H_2_O_2_-stimulated cells, compared to the untreated control cells. However, the increased levels of p–γH2AX by H_2_O_2_ were inhibited in the presence of honokiol (Figure 3(C)). We also investigated the protective effect of honokiol on DNA damage by assessing the level of 8-OHdG, a specific marker of DNA oxidative damage. Figure 3(D) shows that H_2_O_2_ treatment significantly increased the production of 8-OHdG adduct compared to the control group, but pretreatment of honokiol markedly reduced the production of 8-OHdG by H_2_O_2_.

### Honokiol diminishes H_2_O_2_-induced mitochondrial dysfunction

**Figure 4.**
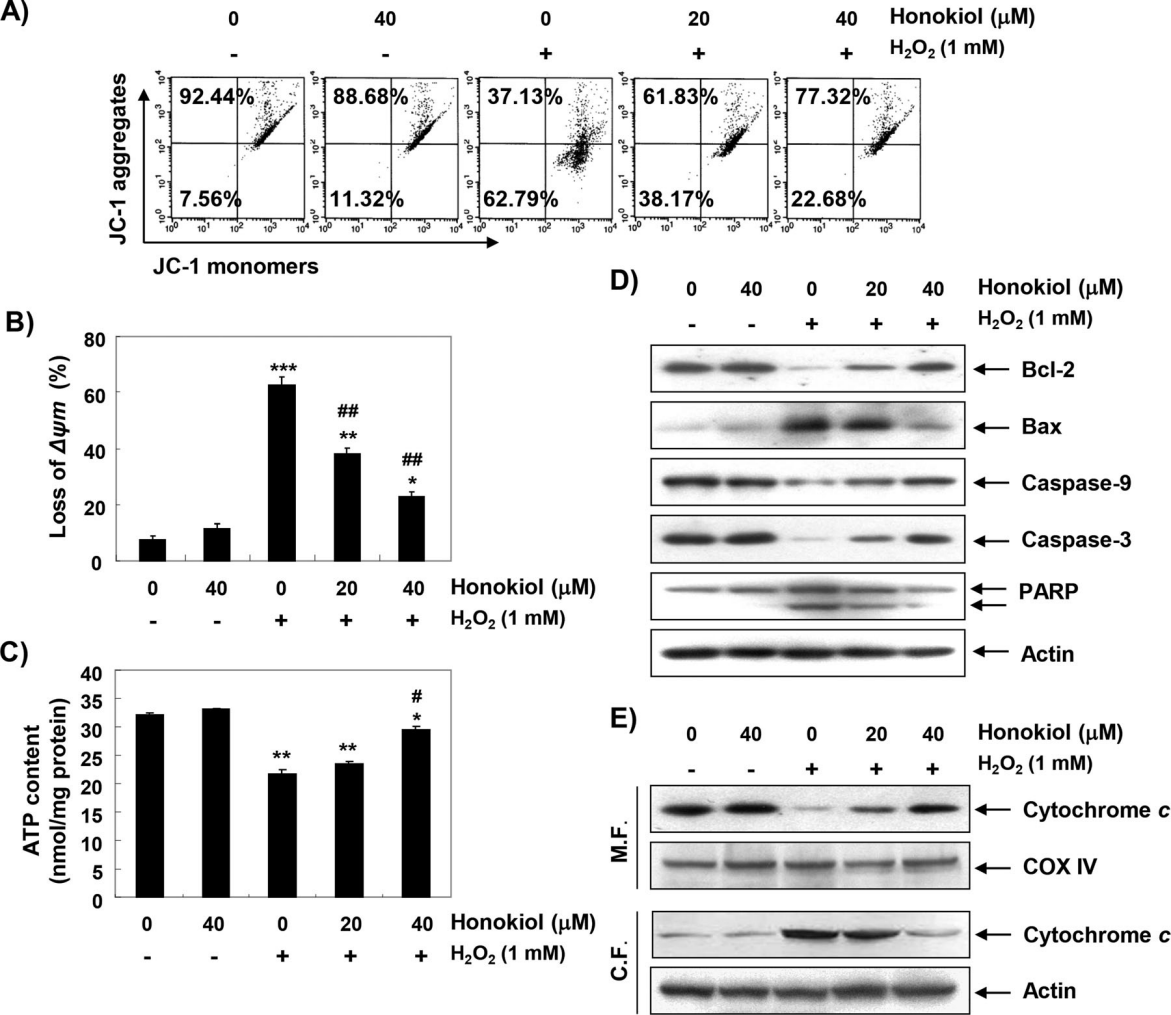
Attenuation of H_2_O_2_-induced mitochondrial dysfunction and changes of apoptosis regulatory proteins by honokiol. Cells were pretreated with honokiol for 1 h, and then stimulated with or without 1 mM H_2_O_2_ for 24 h. (A) The cells were incubated with 10 µM JC-1, and the values of MMP were evaluated by a flow cytometer. (B) The data are shown as mean ± SD obtained from three independent experiments. (C) ATP production was monitored using a luminometer. The results are the mean ± SD obtained from three independent experiments. Statistical analyses were conducted using analysis of variance (ANOVA-Tukey’s *post hoc* test) between groups. *p < 0.05, **p < 0.01 and ***p < 0.001 *vs* control group, ^#^*p *< 0.05 and ^##^*p *< 0.01 *vs* H_2_O_2_-treated group. (D) The cellular proteins were prepared, and the protein levels were assayed by Western blot analysis. (E) The mitochondrial and cytosolic proteins isolated from cells were separated by SDS polyacrylamide gel electrophoresis, and transferred to the membranes. The membranes were probed with anti-cytochrome *c* antibody. Equal protein loading was confirmed by the analysis of cytochrome *c* oxidase subunit IV (COX IV) and actin in each protein extract (M.F., mitochdrial fraction; C.F., cytosolic fraction).

To examine the protective effect of honokiol on mitochondrial dysfunction by H_2_O_2_, MMP and intracellular ATP levels were evaluated. As shown in Figure 4(A,B), changes in ratio of the polarized and depolarized cell populations were observed in cells treated with H_2_O_2_, and the increase in depolarized mitochondrial membrane was about 8 times higher than in the control group. Along with the results, the concentration of ATP in cells exposed to H_2_O_2_ was significantly decreased; however, honokiol could significantly block these changes (Figure 4(C)).

### Honokiol restores H_2_O_2_-induced alteration of the apoptosis regulatory genes

To further investigate the mechanisms of the anti-apoptotic effect of honokiol, we examined the effect of honokiol on H_2_O_2_-induced changes of apoptosis-regulated gene expression. The immunoblotting results show that the anti-apoptotic Bcl-2 protein was significantly down-regulated in H_2_O_2_-treated cells, while the pro-apoptotic Bax protein was up-regulated. Additionally, the expression of pro-caspase-9 and −3 was markedly reduced in H_2_O_2_-treated cells, and the expression of cleaved poly (ADP-ribose) polymerase (PARP) was increased (Figure 4(D)). Further, the expression of cytochrome *c* in H_2_O_2_-stimulated cells was increased in the cytoplasmic fraction, indicating that cytochrome *c* was released from the mitochondria into the cytoplasm (Figure 4(E)). However, these changes by H_2_O_2_ treatment were relatively conserved in the honokiol-pretreated cells.

## Discussion

**Figure 5.**
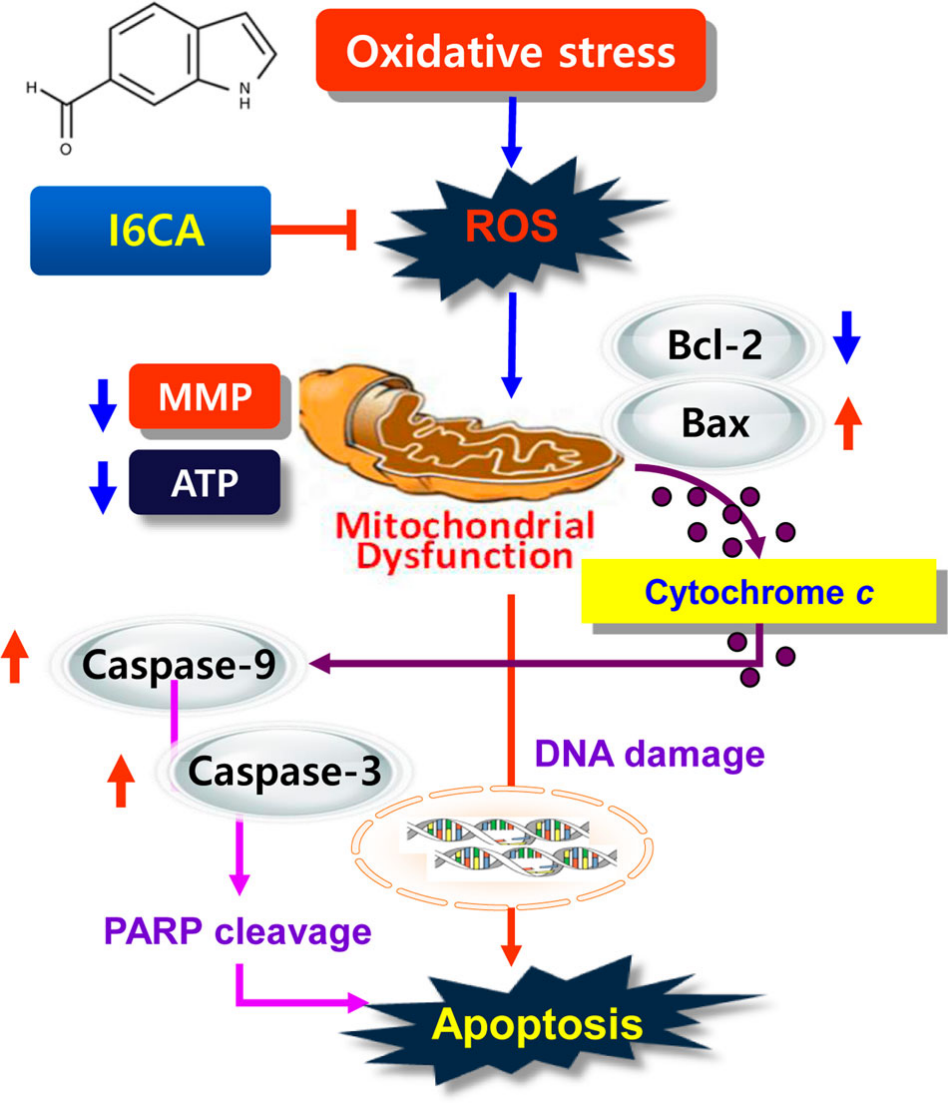
Schematic pathways for the inhibitory effect of honokiol on oxidative stress-mediated DNA damage and apoptosis in C2C12 cells.

As seen in many previous studies, the accumulation of ROS due to the imbalance of ROS production and defense of antioxidant systems of skeletal muscle cells can oxidize important components of the cells ultimately leading to DNA damage and apoptosis (Terrill et al. 2013; Pohjoismäki and Goffart 2017). Such skeletal muscle cell death plays a crucial role in the development of muscle atrophy, and the elevation of ROS levels is related to the degree of skeletal muscle cell damage in atrophic conditions (Zuo and Pannell 2015; Gao et al. 2018). Recently, H_2_O_2_-induced oxidative stress has been shown to induce functional impairment of mitochondria, which can lead to myoblast damage (Wang et al. 2016; Kim and Yi 2018; Ábrigo et al. 2018). Thus, these observations suggest that the prevention of myoblast death by oxidative stress may be a promising strategy to prevent skeletal muscle wasting. Current results have shown that H_2_O_2_ activated DNA damage and induced apoptosis by inducing ROS production in C2C12 myoblasts; however, honokiol significantly prevented H_2_O_2_-induced cytotoxicity, by inhibiting DNA damage through reduction of ROS accumulation. The results indicate that H_2_O_2_-mediated cytotoxicity in C2C12 myoblasts were achieved by inducing ROS-dependent DNA damage. The present finding is summarized in Figure 5.

In inducing excessive ROS-mediated apoptosis, ROS overload causes free radical attack of the membrane phospholipid, which in turn leads to mitochondrial membrane depolarization leading to the loss of MMP, which is considered to be the onset of the intrinsic apoptosis pathway (Rigoulet et al. 2011; Sosa et al. 2013). At the same time, mitochondrial dysfunction promotes abnormalities in the mitochondrial respiratory chain’s electron transport pathways, ultimately interfering with the production of intracellular ATP (Valero 2014; Tian et al. 2017). According to current studies, pre-treatment with honokiol significantly reversed H_2_O_2_-induced loss of MMP and ATP contents. These results are in good agreement with previous studies that show that the protective effects of apoptosis against oxidative stress in various muscle cell models are related to the maintenance of ATP production by the preservation of mitochondrial function (Valero 2014; Tian et al. 2017). Therefore, we consider that the conservation of ATP production due to the retention of mitochondrial membrane function is one possible mechanism of honokiol to preserve the cell survival pathway from oxidative stress.

The signalings for apoptosis initiation vary depending on the stimulus, but are largely divided into the death receptor (DR)-mediated extrinsic and mitochondria-mediated intrinsic pathways. In the extrinsic pathway, the interaction between the death ligands and the corresponding DRs ultimately activates caspase-8. On the other hand, the initiation of the intrinsic pathway requires activation of caspase-9 by the release of apoptogenic factors, including cytochrome *c* from the mitochondria to the cytoplasm (Rigoulet et al. 2011; Tummers and Green 2017). Activation of the initiating caspases, including caspase-9 and −8, ultimately activates downstream effector caspases, including caspase-3/-7, eventually leading to apoptosis. This process is accompanied by degradation of the substrate proteins of effector caspases, such as PARP, as evidence that caspase-dependent apoptosis is induced (Kiraz et al. 2016; Tummers and Green 2017). The activity of the caspase cascade for the induction of apoptosis is regulated by various proteins, including Bcl-2 family members. Among the Bcl-2 family members, anti-apoptotic proteins, such as Bcl-2, are located on the outer mitochondrial membrane to prevent the release of apoptogenic factors, and provide protection by inhibiting the consumption of ATP (Gustafsson and Gottlieb 2007; Kiraz et al. 2016). On the other hand, pro-apoptotic proteins, including Bax, antagonize anti-apoptotic proteins, or translocate to mitochondrial membranes to induce mitochondrial pore formation leading to the loss of MMP, resulting in the cytosolic release of apoptotic factors (Imahashi et al. 2004; Kulikov et al. 2012). Therefore, the balance of apoptotic Bax family proteins to the anti-apoptotic Bcl-2 family proteins serves as a determinant for activating or inhibiting the intrinsic apoptosis pathway. Many previous studies have shown that the induction of apoptosis by H_2_O_2_ in C2C12 cells was associated with a decrease in the Bcl-2/Bax ratio and/or activation of caspases (Siu et al. 2009; Haramizu et al. 2017). Consistent with previous findings, our results also showed that the cytosolic release of cytochrome *c*, and the decreased expression of Bcl-2 and increased expression of Bax observed in H_2_O_2_-treated cells abolished in the presence of honokiol. In addition, H_2_O_2_-induced activation of caspase-9 and -3, and the degradation of PARP, were also significantly blocked by honokiol. In this respect, it is suggested that honokiol inhibits H_2_O_2_-induced apoptosis by decreasing the increase of the Bax/Bcl-2 expression ratio, which means that the reduction reduces cytochrome *c* release from the mitochondria to the cytoplasm. This, in turn, protects the activation of caspase cascade signaling pathway.

In summary, the present study demonstrates that honokiol could effectively prevent H_2_O_2_-induced oxidative stress, mitochondrial dysfunction, DNA damage and apoptosis, through its antioxidant action in C2C12 myoblasts. Honokiol was also able to alter the reduced ratio of Bcl-2/Bax and activation of caspases, which may contribute to the protective effect of honokiol on H_2_O_2_ exposure-induced apoptosis, as shown in the composite scheme in Figure 5. Although the results of this study were performed using a cell line and *in vitro* assays, it may provide important information about signaling molecules protecting muscle cells under oxidative damage.
